# Supplementary material for: An open-source framework for large-scale, flexible evaluation of biomedical text mining systems
Source: J Biomed Discov Collab. 2008 Jan 29;3:1. doi: 10.1186/1747-5333-3-1 (PMC2276192; doi:10.1186/1747-5333-3-1)
Supplement: Additional file 1 — Evaluation platform source code. The additional file contains the software evaluation framework discussed in this paper. The most current release can be downloaded from SourceForge.net [1]. See the accompanying README file for instructions on installation and use. Also included in the distribution is a collection of UIMA wrappers for some commonly used BioNLP tools and annotated corpora. [file 1747-5333-3-1-S1.zip › bionlp_uima_repository-1.0.1/docs/api/src-html/edu/uchsc/ccp/uima/annotation/CCPAnnotationSet_Type.html]

```
001    
002    /* First created by JCasGen Tue Aug 28 16:44:10 MDT 2007 */
003    package edu.uchsc.ccp.uima.annotation;
004    
005    import org.apache.uima.cas.Feature;
006    import org.apache.uima.cas.FeatureStructure;
007    import org.apache.uima.cas.Type;
008    import org.apache.uima.cas.impl.CASImpl;
009    import org.apache.uima.cas.impl.FSGenerator;
010    import org.apache.uima.cas.impl.FeatureImpl;
011    import org.apache.uima.cas.impl.TypeImpl;
012    import org.apache.uima.jcas.JCas;
013    import org.apache.uima.jcas.JCasRegistry;
014    import org.apache.uima.jcas.cas.TOP_Type;
015    
016    /** The annotation set provides a means for arbitrarily categorizing or clustering groups of annotations. Annotations can be associated with multiple annotation groups. Examples of use include, defining Gold Standard annotation sets, and delineating between the use of different parameters during annotation, among others. Each annotation set is associated with a unique ID, a name and a description.
017     * Updated by JCasGen Tue Aug 28 16:44:10 MDT 2007
018     * @generated */
019    public class CCPAnnotationSet_Type extends TOP_Type {
020      /** @generated */
021      protected FSGenerator getFSGenerator() {return fsGenerator;}
022      /** @generated */
023      private final FSGenerator fsGenerator = 
024        new FSGenerator() {
025          public FeatureStructure createFS(int addr, CASImpl cas) {
026                             if (CCPAnnotationSet_Type.this.useExistingInstance) {
027                               // Return eq fs instance if already created
028                         FeatureStructure fs = CCPAnnotationSet_Type.this.jcas.getJfsFromCaddr(addr);
029                         if (null == fs) {
030                           fs = new CCPAnnotationSet(addr, CCPAnnotationSet_Type.this);
031                               CCPAnnotationSet_Type.this.jcas.putJfsFromCaddr(addr, fs);
032                               return fs;
033                         }
034                         return fs;
035            } else return new CCPAnnotationSet(addr, CCPAnnotationSet_Type.this);
036              }
037        };
038      /** @generated */
039      public final static int typeIndexID = CCPAnnotationSet.typeIndexID;
040      /** @generated 
041         @modifiable */
042      public final static boolean featOkTst = JCasRegistry.getFeatOkTst("edu.uchsc.ccp.uima.annotation.CCPAnnotationSet");
043     
044      /** @generated */
045      final Feature casFeat_annotationSetID;
046      /** @generated */
047      final int     casFeatCode_annotationSetID;
048      /** @generated */ 
049      public int getAnnotationSetID(int addr) {
050            if (featOkTst && casFeat_annotationSetID == null)
051          jcas.throwFeatMissing("annotationSetID", "edu.uchsc.ccp.uima.annotation.CCPAnnotationSet");
052        return ll_cas.ll_getIntValue(addr, casFeatCode_annotationSetID);
053      }
054      /** @generated */    
055      public void setAnnotationSetID(int addr, int v) {
056            if (featOkTst && casFeat_annotationSetID == null)
057          jcas.throwFeatMissing("annotationSetID", "edu.uchsc.ccp.uima.annotation.CCPAnnotationSet");
058        ll_cas.ll_setIntValue(addr, casFeatCode_annotationSetID, v);}
059        
060      
061     
062      /** @generated */
063      final Feature casFeat_annotationSetName;
064      /** @generated */
065      final int     casFeatCode_annotationSetName;
066      /** @generated */ 
067      public String getAnnotationSetName(int addr) {
068            if (featOkTst && casFeat_annotationSetName == null)
069          jcas.throwFeatMissing("annotationSetName", "edu.uchsc.ccp.uima.annotation.CCPAnnotationSet");
070        return ll_cas.ll_getStringValue(addr, casFeatCode_annotationSetName);
071      }
072      /** @generated */    
073      public void setAnnotationSetName(int addr, String v) {
074            if (featOkTst && casFeat_annotationSetName == null)
075          jcas.throwFeatMissing("annotationSetName", "edu.uchsc.ccp.uima.annotation.CCPAnnotationSet");
076        ll_cas.ll_setStringValue(addr, casFeatCode_annotationSetName, v);}
077        
078      
079     
080      /** @generated */
081      final Feature casFeat_annotationSetDescription;
082      /** @generated */
083      final int     casFeatCode_annotationSetDescription;
084      /** @generated */ 
085      public String getAnnotationSetDescription(int addr) {
086            if (featOkTst && casFeat_annotationSetDescription == null)
087          jcas.throwFeatMissing("annotationSetDescription", "edu.uchsc.ccp.uima.annotation.CCPAnnotationSet");
088        return ll_cas.ll_getStringValue(addr, casFeatCode_annotationSetDescription);
089      }
090      /** @generated */    
091      public void setAnnotationSetDescription(int addr, String v) {
092            if (featOkTst && casFeat_annotationSetDescription == null)
093          jcas.throwFeatMissing("annotationSetDescription", "edu.uchsc.ccp.uima.annotation.CCPAnnotationSet");
094        ll_cas.ll_setStringValue(addr, casFeatCode_annotationSetDescription, v);}
095        
096      
097    
098    
099    
100      /** initialize variables to correspond with Cas Type and Features
101            * @generated */
102      public CCPAnnotationSet_Type(JCas jcas, Type casType) {
103        super(jcas, casType);
104        casImpl.getFSClassRegistry().addGeneratorForType((TypeImpl)this.casType, getFSGenerator());
105    
106     
107        casFeat_annotationSetID = jcas.getRequiredFeatureDE(casType, "annotationSetID", "uima.cas.Integer", featOkTst);
108        casFeatCode_annotationSetID  = (null == casFeat_annotationSetID) ? JCas.INVALID_FEATURE_CODE : ((FeatureImpl)casFeat_annotationSetID).getCode();
109    
110     
111        casFeat_annotationSetName = jcas.getRequiredFeatureDE(casType, "annotationSetName", "uima.cas.String", featOkTst);
112        casFeatCode_annotationSetName  = (null == casFeat_annotationSetName) ? JCas.INVALID_FEATURE_CODE : ((FeatureImpl)casFeat_annotationSetName).getCode();
113    
114     
115        casFeat_annotationSetDescription = jcas.getRequiredFeatureDE(casType, "annotationSetDescription", "uima.cas.String", featOkTst);
116        casFeatCode_annotationSetDescription  = (null == casFeat_annotationSetDescription) ? JCas.INVALID_FEATURE_CODE : ((FeatureImpl)casFeat_annotationSetDescription).getCode();
117    
118      }
119    }
120    
121    
122    
123
```
